# Stroke-Associating Acute Limb Ischemia Due to the Rupture of a Hydatid Cyst

**DOI:** 10.3390/cimb45030170

**Published:** 2023-03-22

**Authors:** Mihaela Lungu, Violeta Diana Oprea, Andrei Lucian Zaharia, Bianca Stan, Laura Rebegea, Dan Iulian Mocanu, Eva Maria Elkan, Elena Niculet, Ana Croitoru

**Affiliations:** 1 “St. Apostle Andrei” Clinical Emergency County Hospital Galati, 800578 Galati, Romania; 2 Faculty of Medicine and Pharmacy, “Dunarea de Jos” University of Galati, 800216 Galati, Romania; 3 “St. Joan” Pediatric Clinical Emergency Hospital Galati, 800487 Galati, Romania

**Keywords:** ruptured hydatid cyst, anhistous membrane embolism, stroke, acute limb ischemia, anaphylaxis

## Abstract

(1) Background: Hydatidosis, or human cystic echinococcosis, is a zoonotic disease. Endemic in some areas, recently it has an increasing incidence in wider regions, determined by population migration. Clinical features depend on the localization and level of infection: asymptomatic or with signs related to hypersensitivity, organic functional deficiencies, expanding mass effects, cyst infection and sudden death. In rare cases, the rupture of a hydatid cyst causes emboli formation by the residual laminated membrane. (2) Methods: We performed an extensive literature review, starting from the case of a 25-year-old patient presenting with neurologic symptoms relevant for acute stroke, associating right upper limb ischemia. (3) Results: Imaging investigations revealed the source of the emboli as the rupture of a hydatid cyst, the patient presenting multiple pericardial and mediastinal localizations. Cerebral imaging confirmed an acute left occipital ischemic lesion, with complete recovery of the neurological deficit after therapy, while surgery for acute brachial artery ischemia had a favorable postoperative evolution. Specific anthelmintic therapy was initiated. An extensive literature review using available databases revealed the scarcity of data on embolism as a consequence of cyst rupture, highlighting the significant risk of clinicians overlooking this possible etiology. (4) Conclusions: An associated allergic reaction should raise the hypothesis of a hydatid cyst rupture as a cause of any level acute ischemic lesion.

## 1. Introduction

Human cystic echinococcosis (CE), also known as hydatidosis, is a zoonotic disease with various clinical manifestations depending on the localization of cysts, being caused by the larval stages of the cestode Echinococcus granulosus (or other Echinococcus species determined to be of human health concern) [1,2,3].

Although it used to be an endemic disease in some areas, its general incidence has been increasing in the last four decades, due to transcontinental population migration. Generally, a single cyst forms (60–80% of cases), but there are cases presenting multiple cysts located in virtually any organ of the human body [1]. Clinical features are related to the localization and level of infection, not necessarily manifesting in all patients, as there are cases with long asymptomatic evolution. Symptoms mainly result from pressure effects caused by the enlarging cysts or are consequences of their rupture: hypersensitivity/anaphylaxis, organ insufficiency or functional disturbances, obstruction of the biliary ducts and kidneys, bronchi, or cerebrospinal fluid drainage pathways with the appearance of acute hydrocephalus. Multi-organ dissemination involves hepatic, pulmonary, cerebral and cardiac lesions, with long term consequences [1,2,3,4,5,6,7].

From human ingestion of the egg or larvae of the flatworm Echinoccocus, to its clinical manifestations, the evolution may be clinically asymptomatic, even for several decades [3,4,5]. After ingestion, the parasite evolves as a hexacanthic embryo, attaching to the duodenal or jejunal wall and penetrating it, then it reaches the liver through the portal circulation, where the embryos typically remain confined.

However, embryos with a maximal diameter 0.3 mm may overpass the hepatic sinusoids and, through the hepatic vein and the inferior vena cava, reaching the right heart and, subsequently, the lung [2,3]. Other parasites are able to cross the lung barrier and enter the systemic circulation [3,6,7,8,9].

A similar clinical presentation in which the rupture of a hydatid cyst causes the emboli simultaneously in a cerebral artery, triggering a stroke, and in a major artery of the upper limb—with the appearance of its acute ischemia—could not be identified in the available literature. The extensive literature research we performed proved that the embolic ischemia of the peripheral arteries in a limb was reported rarely, similar to the cases of the embolic stroke in patients with a ruptured hydatid cyst.

## 2. Materials and Methods

Starting from an interesting and rare patient case presenting to the emergency room with acute right upper limb ischemia, clinical signs suggesting a stroke and allergic skin lesions, we performed an extensive literature review aiming to identify similar cases, if any, and the recommended clinical and therapeutic approach.

## 3. Case Report

We present the clinical case of a 25-year-old patient who was admitted to the Emergency Department with acute right upper limb ischemia, raising the suspicion of an embolism in the right brachial artery, with sudden onset within the day preceding the presentation to the hospital, while the patient also complained of an associated severe headache.

Inflammatory skin lesions suggestive of an allergic reaction were observed in the cephalic region (edema of the face, rush), associating dyspnea. The patient also had right lateral homonymous hemianopsia and severe occipital cephalea, occurring concomitantly with the other symptoms in the previous 24 h.

From personal medical history, the only significant event was a left brachio-brachial bypass performed in 2015 (following a contusion of the left brachial artery due to a horse bite).

Other observations during the current clinical examination were the underweight status of the patient and painless hepatomegaly.

Lab tests showed a mild hepatocytolytic syndrome, an inflammatory syndrome with serum level of C-reactive protein over 100 mg/L, upper limit of normal (ULN) = 5 mg/mL, erythrocyte sedimentation rate (ESR) = 30 mm/h, leukocytes = 8.44 × 10^3^/µg, eosinophilia = 9% (ULN = 4%). Liver and heart ultrasound evaluations did not detect any pathological changes.

**Figure 1:**
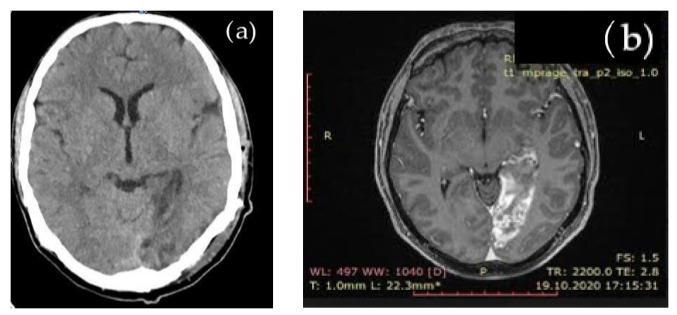
Cerebral imaging: (**a**) native brain CT aspect: left occipital ischemic hypodense lesion; (**b**) brain MRI aspect: left occipital ischemia.

Due to stroke, suggesting symptoms, cerebral native CT and subsequent brain MRI evaluation showed an acute left occipital ischemic lesion, shown in Figure 1.

The brain MRI detected an acute ischemia in the left occipital area, excluding other types of cerebral lesions.

**Figure 2:**
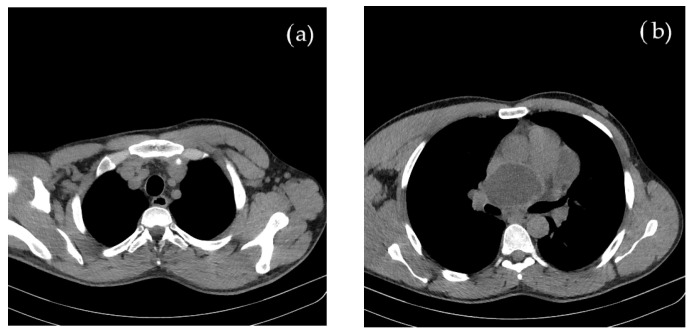
Contrast and native CT of thorax and abdomen shows multiple (**a**) mediastinal and (**b**) pericardial hydatid cysts.

Concomitant onset of limb and cerebral ischemia was suggestive of a common etiology, most probable of cardiac origin. The CT evaluation of the thorax and mediastinum was further performed to investigate the cause of suddenly occurring dyspnea, revealing multiple round, thin-walled lesions, with fluid content (cystic), located both mediastinal and pericardial, suggestive for hydatid cysts; a well-defined cystic formation of 69 mm in diameter appeared on the projection area of the left atrium. Around the postero-superior and apex of the heart, adjacent to the pericardium, three cystic lesions of 80/43/96 mm were identified. In the lower paratracheal region (Barety space level), a cystic formation of 30/26/24 mm was also visible. No other thoraco-abdominal pathological images were detected, as shown in Figure 2.

The symptomatology, imaging and laboratory investigations confirmed the diagnosis of active hydatidosis with pericardial and mediastinal lesions, raising the suspicion that the cysts’ ruptures resulted in acute ischemia in both occipital lobe and right upper limb.

**Figure 3:**
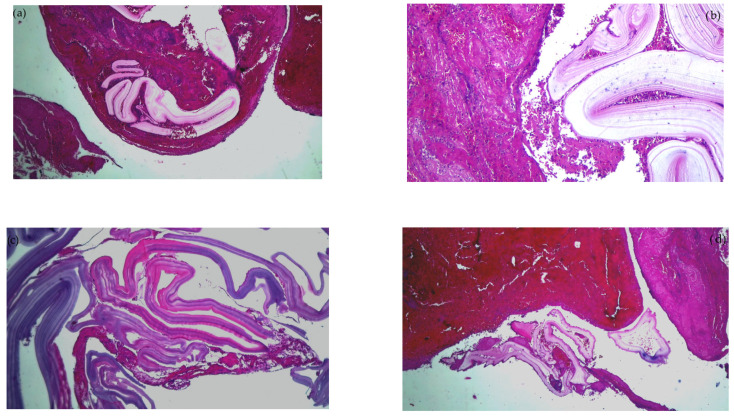
Histopathological examination of the hydatid cyst membrane collected from the brachial artery: (**a**) HE × 40: fibrin clot enclosing fragments of the anhistous membrane; (**b**) HE × 100: anhistous membrane fragment embedded in fibrin-blood clot; (**c**) HE × 40: anhistous membrane fragment; (**d**) Histopathological examination HE × 40: anhistous membrane fragment and blood clot.

**Figure 4:**
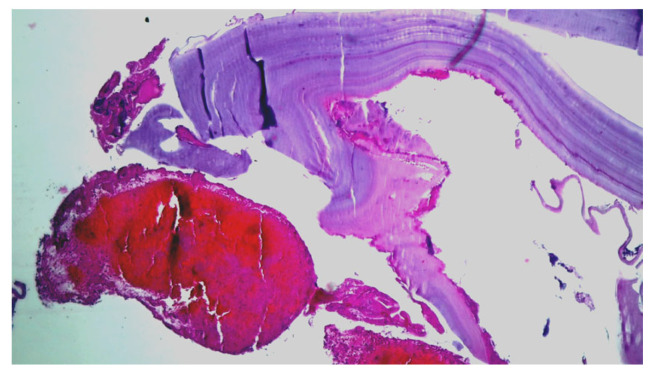
Histopathological examination: HE × 40: anhistous membrane fragment with associated dense inflammatory infiltrate.

Emergency surgery was performed to treat the acute brachial artery ischemia, as the patient showed early signs of motor deficit. A surgical sample fragment suggesting a ruptured hydatid cyst membrane was histopathologically assessed and confirmed the diagnosis of arterial embolism due to a hydatid cyst membrane fragment. The sample tissue was a fibrino-hematic clot with rich granulocytic inflammatory infiltrates, intense eosinophilia, and included multiple fragments of anhistous membrane (avascular, eosinophilic, retractile, laminated) from the structure of the hydatid cyst. The histopathological aspect was suggestive of a hydatid embolism, seen in Figure 3a–d and Figure 4.

Post-surgery, the patient had a favorable evolution, with the restoration of the blood flow in the right brachial artery. This was also followed by the recovery of the hemianopsia, after standard ischemic stroke therapy (without any anticoagulant medication).

The patient started the medical treatment with albendazole and was recommended regular imaging for monitoring of therapy efficacy, and surveillance of the existing pericardial and mediastinal lesions.

## 4. Discussion

The echinococcal cyst contains an inner germinal layer, an acidophilic, acellular membrane, and a host-produced layer of granulomatous reaction. The germinal layer produces brood capsules that bud internally, generating protoscolices through asexual division [2,5,8,9,10,11]. After ingestion, Echinococcus eggs hatch and release embryos in the small intestine that they penetrate, reaching the portal circulation, and from there they can go in practically every organ. The most affected structure is the liver (63%), followed by the lungs (25%), muscles (5%), bones (3%), kidney (2%), brain and spleen (1%) [2,11,12,13,14,15]. Hydatid cysts may develop in different locations: the heart cavities [9,11,12,13], the central nervous system, heart, kidneys, muscles, or mammary glands, ovary, appendix, pancreas, spleen, salivary and adrenal glands, thyroid, scrotum, gallbladder and bones (e.g., the proximal tibial metaphysis and diaphysis) [8,16,17,18,19,20].

Echinococcus hexacanthic embryos can enter the blood stream from the bowels, continuing then toward the right heart and lungs [5,8,15]. Rarely, the parasite can also reach the lungs directly through inhalation of contaminated aerosols. By the path of the lymphatic system passing through the hepatic dome and diaphragm, the embryos can reach the parasternal and intercostal lymph nodes.

Rupture of a hydatid cyst with a proximal location can cause lung inoculation, resulting in secondary hydatid cysts [5,6,7,11]. Secondary lung lesions can also develop due to the rupture of a cyst in the right heart [8,11]. If the embryos extend beyond the lung, they pass into the general blood flow.

A literature search within PubMed/Medline databases based on relevant keywords identified less than 30 published articles, within a broad range of 45 years focusing on any type of ischemia, due to the rupture of hydatid cysts. This scarcity of data is indicative of the rare occurrence of these complications and probably of the underdiagnosing, although they represent a medical emergency which can manifest as the initial materialization of echinococcosis. 

Cardiac hydatid cyst is a rare localization, which occurs in 0.5–2% of cases, compared to liver (65%) and lung (25%) [9,13]. The larvae reach the heart mostly through the coronary system. Rarely, the heart is reached from the intestinal lymphatic tract through the superior vena cava and the inferior vena cava. Hemorrhoidal or pulmonary veins can also be additional routes of dissemination [9,17,21,22,23]. Cardiac CE may be associated with multi-organ dissemination [3,8,9,10,11,12,13]. Cardiac cysts can lodge on tricuspid valves or in the left ventricular myocardium, septum, or atrium. The clinical picture is dependent on the location of the cyst, but the cyst can be discovered incidentally. The hydatid cyst of the left ventricle is frequently located in the subepicardial layers and rarely ruptures in the pericardial space [9,11,14]. In the right ventricle, the localization is frequently subendocardial, the rupture is more frequent, and the intracavitary rupture causes pulmonary emboli [11,12]. Subendocardial cysts can enter the ventricular cavity, rupture and cause sudden death or peripheral embolism [13,14].

Patients may be asymptomatic for long periods of time, but may also have minor, nonspecific complaints. However, in the absence of diagnosis and specific treatment, the risk of fatal complications of CE is very high [9,21]. Ghadhoune et al. [23] presented the unusual case of a 39-year-old patient admitted to the intensive care unit for respiratory distress, associated with a state of shock occurring after a casual heavy meal. The examination showed peripheral signs of shock, significant cervico-facial edema, as well as an urticarial eruption predominantly in the trunk, associating generalized cyanosis, bradypnea at 8 bpm, diffuse sibilant rales during pulmonary auscultation and a pulse oxygen saturation of 82% under 12 L/min oxygen therapy. The diagnosis was of anaphylaxis and ischemic stroke due to rupture of a hepatic hydatid cyst, where the hypersensitivity was indicative for the parasitic underlying mechanism.

Systemic embolism by echinococcus cyst is rare. It is caused by the rupture of a primary left-sided intracardiac cyst and lodgment of the daughter cysts in an arterial bifurcation, commonly that of the femoral artery, thus the literature case reports of lower limb ischemia, but no upper limbs lesions [16,24,25,26].

Patients with peripheral arterial embolism caused by the rupture of an intracardiac cyst generally have multi-organ localizations [15], and the rupture inside the left heart leads to systemic emboli [16]. Embolisms in the arteries of the lower limb are extremely rare. Our extensive literature review only identified few reports on cases of acute ischemia in the femoral or popliteal artery [9,15]; nevertheless, no other case of an embolism in the upper limb was identified (probably due to anatomic characteristics of limb vascularization).

The embolism in the brachial artery is a vascular surgery emergency, the rapid intervention allowing the resumption of the arterial flow interrupted by the embolus. This is commonly caused by thrombi, which requires rapid initiation of systemic anticoagulation. However, this was not the case for our patient, where the Doppler suggested a different etiology. We did not find any reports of ischemia of upper limbs triggered by hydatid cysts rupture, but Di Bello and Menéndez reported eight cases of acute embolism of the lower limbs by an echinococcus cyst [27]. Ozer et al. [28] presented their experience on two cases of left heart cysts, revealed by a systemic embolic event.

Multiple intracerebral hydatid cysts have been described, as well as cerebral echinococcal embolism, with a cardiac starting point in the left ventricular myocardium [19,20]. Cerebral cysts are frequently supratentorial, in the distribution territory of the terminal branches of the middle cerebral artery. They can cause neurological signs, intracranial hypertension and acute hydrocephalus, requiring surgical treatment where possible [19,20]. The incidence of cerebral hydatid cysts is 1–2%, and it is associated with secondary lesions in other organs, including the spinal cord [17,26]. The usual manifestation of an intracranial hydatid cyst is intracranial hypertension, which may be associated with hemiparesis, seizures, visual disturbances, or altered levels of consciousness. The cerebral hydatid cyst develops in soft tissues, usually growing without resistance, thus forming a single spherical vesicle which may reach a considerable size before manifesting its presence clinically. Cerebral hydatid cysts should be regarded as malignant because of their location and the risk of irreversible damage, so an early diagnosis is important [21,26,27,28,29,30].

Regarding cerebral embolism, with the appearance of an ischemic stroke, the cases cited in the literature are extremely rare [12,14,21,23,31]. Acaturk et al. presented a case of ischemic stroke, secondary to an embolism, from a cardiac hydatid cyst in a 15-year-old boy [12]. Similar stroke cases were reported by Singh et al. in 2003 [18] and Maffeis et al. in 2000 [11].

Even more infrequent are cases where the stroke due to a hydatid cyst rupture is associated with ischemia of other territories. We identified only very few case reports, one being a case of a surgically treated ruptured left ventricular hydatid cyst, presenting with acute stroke, that was later complicated by distal aortic embolism due to perioperative dislodgement of the germinative membrane, so stroke was only followed by a surgical complication [31].

Ischemic stroke of unusual etiology was rarely reported. A retrospective study from the Barcelona Stroke Registry [32] showed that it only represents up to 6% of the total of cerebral infarctions. Young patients with this diagnosis presented a lower in-hospital mortality rate, had no symptoms at discharge but needed a longer hospital stay more frequently. It is important to distinguish these unusual etiology strokes in young patients from the common subtypes, as they require a different approach and have distinct outcomes with better prognosis.

Hydatidosis is very rarely associated with other types of neurological manifestations outside stroke. A case of Kounis syndrome triggered by a ruptured hepatic echinococcal cyst, complicated by anaphylactic shock and syncope, with ECG alterations as a manifestation of anaphylaxis, was communicated by Mirijello et al. [21].

### 4.1. Paraclinical Diagnostic Tools

Paraclinical examination is essential for the diagnosis of hydatidosis. The diagnosis of hydatid infection is complex, requiring multiple paraclinical investigations, in addition to the clinical examination [24].

#### 4.1.1. Imaging

Radiography, ultrasound, CT or MRI usually establish the diagnosis, showing specific images that are suggestive of the etiology. However, there are situations in which the differential diagnosis is required with an amoebic abscess, a congenital cyst or even with malignant processes, for the confirmation specific serological tests to be useful. MRI can spotlight the presence of detached parasitic membranes, a sign of degeneration and/or rupture of the cyst. These membranes are seen floating within the cyst and appear dark on both T1- and T2-weighted images [24,29]. An external cyst rupture appears when both the wall of the cyst and the capsule of the organ harboring it rupture. Imaging is also of great value during monitoring of medical therapy efficacy.

Cardio-pericardial hydatid cysts, similar to those of our patient, are usually visible in the majority of plain-chest radiographic findings [28,33,34,35,36,37]. The typical image is that of an addition that is well limited, having clear edges. It forms one body with the cardiac shadow, from which it makes it difficult to distinguish. In the presence of cardiomegaly, the diagnosis becomes less obvious, as nothing suggests the hydatidosis. ECG is also a valuable routine procedure for the diagnosis of cardiopericardial hydatidosis [29,35,36]. Subepicardial ischemia is seen in the majority of cases and some patients may have an ECG showing a Q-wave of pseudonecrosis. Other important diagnostic tools are echocardiography, cardiac computed tomography (CT) and magnetic resonance imaging (MRI), coronary arteriography.

Imaging (cerebral and chest CT, brain MRI) in our patient were highly relevant and suggestive for hydatid cysts. Clinical manifestations of double ischemia were suggestive of a cystic rupture.

Early recognition and prompt addressing of ischemic complications of ruptured hydatid cysts could be life-saving. Ruptured cardiac cysts should be suspected in young patients coming from an animal farming background, presenting with acute limb ischemia and/or if they have an embolectomy material resembling a germinative membrane [37,38,39,40,41,42].

#### 4.1.2. Biomarkers and Molecular Biology Characterization

In hydatidosis, the immune response depends on the patient’s age, location, number and size of cysts, quality of cyst membranes (thickened, cracked, calcified) and the appearance of hydatid fluid (clear or presenting an overlapping bacterial infection) [42,43,44]. Hypereosinophilia has little value because of its lack of specificity and its fickleness during the course of the disease.

Serological testing in hydatid disease was demonstrated to have a limited diagnostic accuracy, with reported sensitivities and specificities of 60–90%, depending on the cyst localization. Recommended blood tests should include serum C-reactive protein (CRP), estimated sedimentation rate (ESR) and complete blood count (which, however, might only show an eosinophilia) [42,43,44,45,46,47]. Although they may have poor diagnostic accuracy, in some selected cases antigen tests, ELISA, could be used, as well as indirect hemagglutination and complement fixation tests (sensitivities of 25–56% in extra-hepatic disease).

Other useful lab findings, where available, could be provided by enriched antigen and recombinant antigen tests (increasing the diagnostic accuracy, demonstrating sensitivities of over 90% on selected cases). Some protein biomarkers and DNA-based detection methods, such as quantitative or nested PCR assays, could provide better diagnostic accuracy, but are not widely available as routine tests.

The serologic tests for the management of hydatid disease are important in the long run, in order to detect the recurrence or secondary dissemination after surgery, being especially relevant when the tests revert to positive after being negative for a period of time [48,49,50,51,52].

The level of serum-specific antibodies is very low (or absent) in patients with intact cysts, especially in those with old, calcified lesions. However, even for intracavitary rupture, serologic tests are sometimes negative, whereas unspecific hypereosinophilia is frequently found. The rupture of a cyst is accompanied by a significant increase in antibody titer, while in recurrent infections there is no significant variation.

Human Echinococcus granulosus infestation causes increased production of serum immunoglobulins, with specific IgG, IgM, IgE and IgA antibodies.

IgG is increased in all current infestations and in all locations, and may persists for a variable period after cyst ablation. The antibody titer is determined by ELISA (enzyme-linked immunosorbent assay) lab test, and the results must be analyzed in correlation with the clinical diagnosis; a negative result does not exclude hydatidosis. In a clinically relevant context, the test should be repeated every 2–4 weeks, several times.

Significant cross-activity with Taenia Solium has also been reported [20].

In human cystic echinococcosis, the immune response is the result of the permanent diffusion of antigenic substances through the cyst walls. These exchanges are the basis of the structural features of the cystic membrane, which can be intact, cracked, thickened or calcified [35,36,42,48,49].

False negative results are caused by the smaller size and integrity of cysts. Lower antibody titers are detected in small hydatids with thickened fibrous capsules, calcification of cysts after the death of the parasite (or causing its death), infection of the cyst with pathogenic germs by biliary cystic fistulas, compromised immune status of the patient. Cerebral, pulmonary or ocular localization result in lower antibody titers [8,19,20].

Eosinophilia and blood leukocytosis are additional diagnostic keys, but their absence does not exclude echinococcosis. Serological methods are those that allow an indirect indicative diagnosis [24,42].

In our patient case, positive unspecific lab tests (biologic inflammatory syndrome, mild eosinophilia) completed the clinical assessment and the imaging. No specific antibodies were evaluated as emergency surgery with etiologic confirmation was performed, so no further serology was necessary.

Molecular biology characterization of Echinococcus granulosus allowed for the identification of various species and genotypes, using studies on nuclear and mitochondrial genes: DNA amplification and sequencing, polymerase chain reaction (PCR) analysis such as rDNA internal transcribed spacer 1 (ITS1-rDNA), analysis of the mitochondrial NADH dehydrogenase subunit 1 (NADH-1) gene or PCR-restriction fragment length polymorphism (PCR-RFLP), primers specific for the gene sequence of the enzyme cytochrome oxidase subunit I (COX1), etc. As a result of these research studies, the *E. granulosus* complex has been grouped into five different species: *E. granulosus sensu stricto* (s.s.) (genotypes G1 and G3), *E. equinus* (G4), *E. ortleppi* (G5), *E. canadensis* (G6–G8 and G10), and *E. felidis*, all being epidemiologically and geographically distinct. Data showed that *E. granulosus* s.s. is endemic in all of the Mediterranean basin and is the main causative agent of CE in humans [53,54].

Expanding the existing sequence data on the genetic diversity of *E. granulosus*, it is necessary to better understand the biology, ecology and molecular epidemiology of this parasite, as we have evidence that different genotypes would probably exhibit different antigenicity, transmission profiles, pathological consequences and different sensitivity to chemotherapeutic agents. Therefore detailed characterization may be required whenever the evolution and therapy outcomes are not optimal. Proteomic studies of helminth Echinococcus products have been particularly valuable for the identification of proteins involved in host—parasite relationship. A species-specific set of proteins could define molecular markers for parasite diagnosis [53,55]. Until now we have identified ten distinct genotypes of the human CE-causing agent *E. granulosus* sensu lato (s.l.), designated as G1–G10, described worldwide on the basis of genetic diversity related to nucleotide sequences of the mitochondrial NADH 1 and COX1 genes, and being associated with distinct intermediate hosts [53,55].

*E. granulosus* genotypes currently recorded in the GenBank database allow for a detailed examination of the genetic variation and differentiation of the *E. granulosus* genotypes across the world. The genotype G1 is responsible for most human CE worldwide (88.44%) and has the most cosmopolitan distribution [54,55]. The World Health Organization (WHO) identified animal vaccination with an *E. granulosus*-recombinant antigen (EG95) as having encouraging prospects for prevention and control. The concept to develop Eg95 was initiated on the basis of identification of individual oncosphere components that stimulate host-protective immune responses in sheep [55,56].

The treatment of hydatid cysts is an emergency for cardiac/cerebral/pulmonary lesions due to the high risk of mortality and dissemination [21,24,43,48,57,58,59,60,61,62,63,64]. Surgery or PAIR (puncture, aspiration, injection of protoscolicidal agent and reaspiration) are required for cystic lesions. Embolectomy is recommended in all situations of acute arterial obstruction [42,43,44,45,46,58,59,60,61,62,63]. For better treatment outcomes, surgery should be completed with medical therapy comprising benzimidazole drugs (albendazole +/− praziquantel is considered the standard, aiming for scolicidal and anti-cystic activity) [43,46,47,48,49,50,51,52]. The follow-up to detect the appearance of new lesions is mandatory [42,57,58,59,60].

In a recent systematic review, relevant data on genotype and species identification in humans were obtained from 29 European countries, identifying Southern and Southeastern Europe (including Romania) as the hotspots of human CE caused by *E. granulosus* s.s., in areas where sheep breeding is done on farms [65] (see Table 1).

Molecular biology characterization of Echinococcus is essential in further investigating differences in the epidemiology, morbidity and mortality due to this parasitic infection in humans. This will significantly impact the diagnosis and efficient therapy of CE, but also provide important insights for a more accurate clinical assessment and prognosis.

## 5. Conclusions

CE is a neglected zoonosis, having an important socio-economic impact, with a worldwide geographical distribution, where the Mediterranean basin is considered an important endemic area. Hydatid cysts of the heart are very rare and are characterized by clinical latency. Rupture with the left-sided chambers may cause systemic emboli with various localizations, but embolization to brain or a limb artery is very rare [14,18,52,57,58,59,60,61,62,63,64].

We reported a case that is unique due to two features:

- A never-reported embolism in the right brachial artery with anhistous membrane, histopathologically was confirmed. We found no similar report in the available literature.
- Concomitant cerebral embolism with an acute ischemic stroke, in a patient without other detectable cardiovascular risk factors, was recorded.

Both installed simultaneously in the context of allergic cutaneous phenomena and dyspnea. Emergency embolectomy allowed for the complete resumption of blood flow from the brachial artery, while maintaining a normal functionality of the upper limb. Specific medical therapy with albendazole was initiated. The evolution of the cerebral embolism was favorable, the patient fully recovering the neurological deficit. Moreover, allergic phenomena were limited with no threat to the patient’s life.

Peripheral arterial embolism in the brachial artery is an extremely rare clinical situation, the literature citing only few cases involving the femoral or popliteal arteries [4,10,16,29,31,33,35,36,59]. Additionally, we identified very few reports of stroke due to embolism after a ruptured hydatid cyst [12,14,17,18,21,22,30,61,62].

The onset of neurological signs occurring simultaneously with peripheral embolism, in the context of suddenly installed allergic skin manifestations, are a solid argument that the rupture of a hydatid cyst caused embolism with anhistous membranes in our patient, ischemia in the right brachial artery and left posterior cerebral artery (stroke being confirmed by CT and MRI) associated with anaphylactic phenomena [51,62,63,64].

The biological differences between the species and genotypes have the potential to affect the transmission dynamics of the parasite, thus requiring modification of methods used in disease-control initiatives, while affecting intermediate host susceptibility.

## Figures and Tables

**Table 1:** Human CE cases molecularly confirmed in Europe (2020–2021). Data from Casulli A. et al. systematic review, 2022 [65].

2022 Systematic Review	*E. granulosus* s.s.			*E. canadensis*			*E. ortleppi*
	Genotype G1 (%)	Genotype G3 (%)	Ungenotyped (%)	Genotype G7 (%)	Genotype G10 (%)	Genotype G6/G10 (%)	Genotype G5 (%)
CE cases circulating Europe	40.3%	10.8%	48.9%	98.3%	0.85%	0.85%	1.5%
CE cases identified Romania	97.4%		-	2.6%			-

## Data Availability

Further medical data on the case report can be obtained from the main authors upon reasonable request.
